# Remote sleep and memory assessments in older adults with mild cognitive impairment and dementia: *Recruitment, retention, and completion of home‐based study tasks*

**DOI:** 10.1002/alz.087966

**Published:** 2025-01-09

**Authors:** Victoria Grace Gabb, Jonathan Blackman, Hamish Morrison, Bijetri Biswas, Haoxuan Li, Nicholas Turner, Alan Whone, Elizabeth Coulthard

**Affiliations:** ^1^ University of Bristol, Bristol United Kingdom; ^2^ North Bristol NHS Trust, Bristol United Kingdom; ^3^ North Bristol NHS Trust, Southmead Hospital, Bristol United Kingdom; ^4^ Bristol Medical School, University of Bristol, Bristol United Kingdom

## Abstract

**Background:**

Poor sleep is associated with neurodegenerative diseases underlying dementia and mild cognitive impairment (MCI), including Alzheimer’s disease (AD) and Lewy body disease (LBD). Performing assessments within clinical or laboratory settings may influence validity, however feasibility of home sleep and memory assessments in this population is currently undetermined. This study aimed to identify whether remote home‐based sleep and memory research including wearable technology was feasible in older adults with MCI and dementia.

**Method:**

Adults aged over 50 with MCI or dementia due to AD or LBD and matched controls (HC) were asked to complete 8 weeks (55 nights) of continuous wrist actigraphy and daily sleep diaries via a mobile application. During one of these weeks, participants were also asked to collect 10 serial saliva samples for cortisol and melatonin assays, wear a wireless electroencephalography (EEG) headband during sleep for 7 nights, and complete 6 short verbal memory tests via videoconferencing. Feasibility metrics on recruitment, retention, data completion, and study support were collated.

**Result:**

***Recruitment***: 129 individuals were potentially eligible during pre‐screening. 44 (34.1%) participants consented to take part. 41 participants (AD = 10, LBD = 11, HC = 20; mean age 71.1, range 57‐81; 22.0% female) met screening criteria and were enrolled. Barriers to recruitment included concerns about ability to complete study tasks, impact on sleep from study assessments, and unfamiliarity with digital technology. ***Retention***: 40/41 (97.6%) participants completed the study. 1 participant withdrew post‐baseline due to personal circumstances. ***Data completion***: Across tasks, data completion rates were high and similar across subgroups. 97.3% (389/400) of saliva samples, 90.0% (144/160) of verbal memory tasks, 93.5% (2,056/2,200) of sleep diaries, 97.0% (2,133/2,200) nights of actigraphy, and 91.4% (256/280) of EEG recordings were completed. Reasons for missing data included technical issues, scheduling difficulties (work/caring commitments), illness, and forgetting to complete or difficulty understanding tasks. ***Study support***: 10 participants (AD = 3, LBD = 7) reported receiving external support (partner or relative) during the study. Regular researcher support was provided via email, video calls, and scheduled SMS/email/in‐app reminders.

**Conclusion:**

Remote, technology‐supported sleep and memory research is feasible in older adults with MCI/dementia and could contribute towards longitudinal, naturalistic, and convenient sleep and memory profiling.

